# Kalgal Burnbona: An Integrated Model of Care Between the Health and Education Sector

**DOI:** 10.5334/ijic.7745

**Published:** 2024-05-02

**Authors:** Santuri Rungan, Huei Ming Liu, Jennifer Smith-Merry, John Eastwood

**Affiliations:** 1 Croydon Health Centre, 24 Liverpool Road, Croydon, NSW, 2132, Australia; 2 Menzies Centre for Health Policy, University of Sydney, Australia; 3 The George Institute for Global Health, University of New South Wales, Australia; 4 Centre for Disability Research and Policy (CDRP), Susan Wakil Health Building (D18), Camperdown Campus, The University of Sydney, NSW, 2006, Australia; 5 School of Population Health, University of New South Wales, Kensington, NSW, 2050, Australia; 6 Sydney Local Health District, Level 11, King George V Building, Missenden Road, Camperdown, NSW, 2050, Australia

**Keywords:** School-based integrated care, School-based health care, health promoting schools, National Children’s Mental Health and Wellbeing Strategy, partnerships between health and education, Healthy Homes and Neighbourhoods (HHAN)

## Abstract

**Introduction:**

Kalgal Burnbona is a framework developed for applying school-based integrated care (SBIC) across Sydney Local Health District (SLHD).

**Description:**

Kalgal Burnbona is an innovative and integrative framework developed to provide holistic, integrated, multidisciplinary child and family centred care to school-aged children from priority populations within SLHD, such as those belonging to the Aboriginal community. The expected outcomes include improved health, behavioural, education and social outcomes. This article contextualises the development of the Kalgal Burnbona framework from its beginnings as a pilot site called Ngaramadhi Space (NS) within the Healthy Homes and Neighbourhoods (HHAN) initiative, through to its evolution to an integrated partnership between the New South Wales (NSW) health and education sector. An example of how the framework can be implemented in other settings within SLHD is described.

**Discussion:**

A tiered approach to integrated care across SLHD is postulated based on evidence from a mixed methods evaluation of NS and in line with the Rainbow Model of Integrated Care (RMIC). Kalgal Burnbona is an example of a community-driven response through collaborative partnerships to improve health, education and social outcomes. The framework described provides structure for multisector teams to work within, recognising that each community and school has its own history and needs.

**Conclusion:**

The Kalgal Burnbona model can be scaled up to serve a wider network of students across SLHD. The initial successes of the model, which include improving access and engagement for children with unmet physical health, mental health and social needs while being accepted by communities provide evidence for policy changes and advocacy that centre on collaborative cross-sector partnerships.

## Introduction

The intertwined relationship between health and education has been well documented [1,2,3,4,5,6]. Schools are an under-utilised community resource and are an important and convenient way for children to access health services [2,7,8].

School-aged children (5–18 years) frequently have unmet physical health and mental health needs due to poor access to health services with a plateauing of global health outcomes in this age group [9,10,11]. Herein lies a need to develop novel, innovative and integrative strategies to improve the health and wellbeing of these children [10]. The international literature shows that school-based healthcare (SBHC) models have been widely adopted, presenting a potential solution for improving access and engagement with health services [2,12,13,14,15,16]. While such models vary considerably across different sites, in general most provide services based on the needs of the community and can include medical assessments, immunisations, counselling and chronic disease management [13,17,18]. Although evaluating these models of care is difficult, due to inherent heterogeneity, they have demonstrated increased access to care, improved health and education outcomes, high levels of student and parent satisfaction, and cost effectiveness [8,12,13,18].

In Australia, children experience poor access to healthcare [11,19,20]. These disparities are more marked in Aboriginal children who experience inequitable education, socio-economic status, physical health, mental health and wellbeing outcomes [21,22,23,24,25,26]. As our understanding of the impact of the Covid 19 pandemic on the widening of these disparities grows, so too has interest in partnering with schools to deliver healthcare across Australia [7,27,28,29,30,31]. While there are several SBHC models across Australia, these have been developed independently of each other without clear frameworks for implementation in different settings or guidelines to inform scaling up [27,32,33].

**Figure 1:**
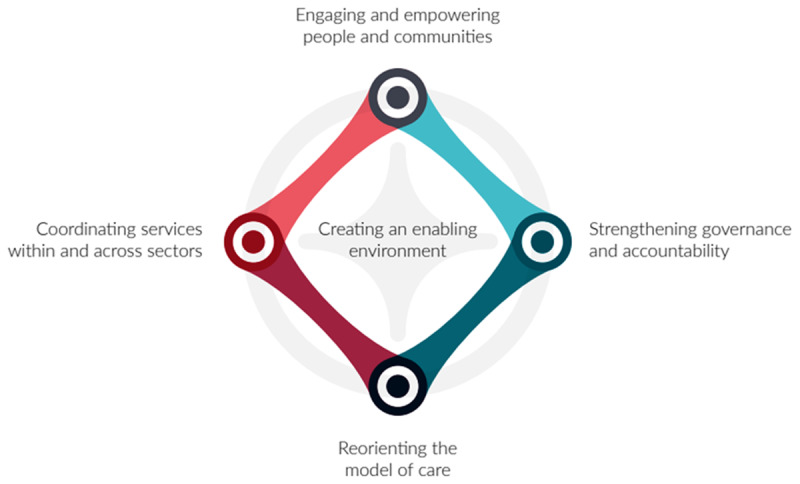
The five interdependent strategies of the WHO Framework on integrated people-centred health services (IPCHS) [37].

In this article we describe Kalgal Burnbona. Kalgal Burnbona is a framework developed for applying school-based integrated care (SBIC) across Sydney Local Health District (SLHD). The term SBIC has been adopted for this framework, rather than SBHC, to emphasise the importance of cross-sector collaboration and partnerships in developing ‘integrated care’. Integrated care refers to the joining together of the various parts of a health system to optimise care with a strong focus on community empowerment [34,35,36]. These underlying principles have been described in the Alma Ata Declaration on Primary Health Care (1978) and the framework for ‘Integrated People-Centred Health Services (IPCHS)’ (Figure 1) [37,38]. Over time there have been inconsistencies and overlap with the definition of ‘integrated care’ with other terms such as ‘patient-centred care’, and ‘coordinated care’ [39]. With integrated care, the emphasis is on patient involvement and collaboration with healthcare providers, as well as meaningful and lasting partnerships across sectors [40]. The Rainbow Model of Integrated Care (RMIC) was developed to describe and evaluate the overarching and multidimensional concepts that inform integrated care [40]. As such, we have attempted to align the Kalgal Burnbona framework with the principles of the RMIC.

Overall, the purpose of Kalgal Burnbona is to provide holistic, integrated, multidisciplinary child and family centred care to school-aged children from priority populations such as Aboriginal children. The expected outcomes of this intervention are to improve health, behavioural, education and social outcomes. This article will contextualise the development of the Kalgal Burnbona framework from its beginnings as a pilot site called Ngaramadhi Space (NS) through to its evolution to an integrated partnership between the New South Wales (NSW) health and education sector that aligns with the RMIC [2,41,42]. Furthermore, we present a case study to exemplify how the framework can be adapted in a new setting within SLHD.

## Historical and Social Context

SLHD is characterised by a diverse and growing population. In 2016 the population of SLHD was 640,000, a 21% increase from 2006. The expected growth rate between 2016–2036 is 40% with an expected population of almost 1 million people by 2036 (43). Children aged 0–17 years (2016) form around 32% of SLHD’s population. Around 1.1% of the population identify as being Aboriginal while 45% were born overseas and 58% speak a non-English language at home [43]. With an 11% prevalence of mental health problems in preadolescent Australian children it can be expected that with this population growth there will be an increase in paediatric-related mental health concerns [44]. Furthermore, mental health issues in children have been compounded by the Covid 19 pandemic with negative impacts emerging on learning, friendships and family relationships, and mental health [28,29,45,46].

Amongst the Aboriginal community, health is connected to ‘Social and Emotional Wellbeing’ (SEWB) [26,47,48]. This is a holistic term that incorporates the significance of physical, mental and emotional health to social, spiritual and cultural wellbeing [24]. To understand the higher prevalence of childhood behavioural disorders in Aboriginal children there is a need to understand how historical, social and cultural factors influence the diagnosis of such disorders [21,49]. Western ideology remains the predominant landscape for describing childhood behaviour and related disorders thereof. Aboriginal life ways and beliefs about the role of children within communities differs from Western culture. For Aboriginal communities, children are active participants in community life and learn through experiences and developing a spiritual connection to the land [50,51,52]. Colonisation disrupted these life ways leading to disconnection from people, place and land [24,50,52]. Western beliefs around behavioural norms were imposed upon Aboriginal people with divergence from these norms considered to be a deviance or a disorder [53,54]. Ongoing and systemic injustices as well as psychological trauma have perpetuated the high incidence of childhood behavioural disorders diagnosed in Aboriginal children and young people [24,55]. For the Aboriginal community, a holistic approach to understanding a child is needed where a ‘whole of child’ and ‘whole of family’ approach is taken [47,56]. The SBIC approach of co-location and integration of health, education and social services is valued by the Aboriginal community with partnerships among services seen as an effective way to maximise limited resources [57,58].

## Kalgal Burnbona

### Early Beginnings

Kalgal Burnbona means ‘to surround family’ in the Dharawal language of the local Aboriginal community. The integrated model of care that informs the framework was co-designed with schools, community paediatrics and the Aboriginal community. The model of care involves a multidisciplinary team made up of members from the health, education and social care sector. The aim of the model is to improve access and engagement to health services for school-aged children and their families by providing comprehensive and holistic assessments and follow up of students [4,12,59]. The Kalgal Burnbona framework has grown out of the Healthy Homes and Neighbourhoods (HHAN) integrated care initiative, where priority schools were strategically identified as potential sites for multiple services to be co-located [60,61]. Out of this process Yudi Gunyi School (YGS), which is a ‘School for Special Purposes’ (SSP) catering for students experiencing problematic externalising behaviour in a mainstream school setting, was identified as a priority school [62,63]. It was at this school that an initial pilot site called Ngaramadhi Space (NS) was established in 2016 [63].

A quantitative evaluation of NS showed that the model of care improved access to healthcare with students having multiple unmet physical health, mental health and social needs. After the multidisciplinary model of care was delivered, behavioural improvements as measured by pre- and post- Strengths and Difficulties Questionnaire (SDQ) teacher reports were noted [64]. A qualitative evaluation of NS and how it could be implemented at other sites showed that SBIC models were effective in engaging and empowering people and communities. The model of care was community-driven and delivered in a culturally-safe manner. Participants identified SBIC as improving access to health care which led to positive outcomes for students and families. SBIC was described as creating ‘connection’ between students and the community with beneficial effects on staff wellbeing (in print (IJIC)). Potential strategies that were identified for implementing SBIC models included community consultation and co-design, building multidisciplinary teams with new competencies and roles e.g. linkers and coordinators, collaborative and shared leadership, and alignment of operational systems while maintaining a balance between structure and flexibility. Furthermore, the need for high-level and ongoing collaboration across sectors and with communities was highlighted (in print (IJIC)).

### Integrated Systems Approach

Statewide interest in the NS model of care and the evaluation findings led us to consider what scaling up would look like. Here, we propose a framework called ‘Kalgal Burnbona’ for implementing SBIC within SLHD.

**Figure 2:**
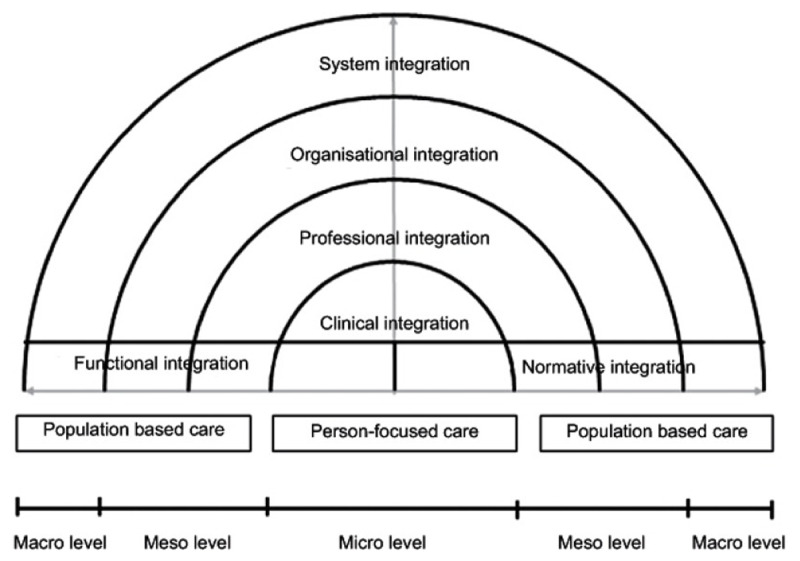
Conceptual framework for integrated care based on the integrative functions of primary care [65].

The Kalgal Burnbona framework was developed with consideration of the RMIC (Figure 2) [40]. The RMIC presents a holistic vision of person-focused and population-based care alongside the dimensions of integrated care [65]. Person-focused care relates to a biopsychosocial perspective of health with a shift from clinician-centred preferences to those of individuals [37,66]. This concept aligns with Aboriginal concepts of SEWB and the importance of taking a holistic approach to health [24,57]. Population-based care considers the wider social determinants of health such as political and economic factors [39]. In developing the Kalgal Burnbona framework, integration of care was considered at the macro (system) level, the meso (organisational) level and the micro (clinical) level [65]. In practice, the pilot site for Kalgal Burnbona, NS, was built from the ground-up mainly utilising meso and micro level partnerships so these aspects will be described first in the following sections.

#### Meso Level: Professional Integration and Organisational Integration

At a meso level two levels of integration are described in the RMIC framework [65]. These include professional integration and organisational integration [39,67]. Professional integration refers to partnerships between professionals within (intra) and between (inter) organisations [39]. Organisational integration refers to how well services are produced and delivered in a linked-up fashion [67].

Professional integration requires a collective belief by various professionals to work in a coordinated way and to share the responsibility for successful implementation of the model including accountability, problem solving and decision-making [68,69]. In developing NS, key stakeholders from the school (school principal, school director, networked specialist facilitator, school counsellor), health (director of community paediatrics, paediatrician, youth health manager, youth health nurse, psychiatrist), social sector (HHAN project manager, director of social work, social worker) and Aboriginal community formed a ‘leaders committee’ to discuss the aims of the model and how it would be delivered [63,70]. Roles and responsibilities were later formalised in a Memorandum of Understanding (MOU). The Aboriginal community were integral in providing cultural input, ongoing direction and governance over the initiative. This led to the formation of a separate ‘community reference committee’ called the Wouwanguul Kanja committee [63,70,71]. The Wouwanguul Kanja committee developed a ‘terms of reference’ that detailed the committee’s role, frequency of meetings and decision-making processes. In forming these two committees, oversight over the program was provided, which in turn formed the basis of professional and organisational integration.

To scale up the SBIC model to other sites, community consultation and collaboration is required to improve the quality and efficiency of service delivery. We propose that two types of committees are required to provide organisational structure and service integration. The first is a an overarching ‘leaders committee’ to align the vision and goals of the initiative with processes, oversee its evaluation, secure financing, provide governance, and manage problems as they arise [69]. In addition, a second ‘community reference committee’ is required for each cluster of schools to manage community-level issues.

Professional integration can be promoted by forming professional networks where knowledge is shared [72]. In scaling up Kalgal Burnbona, regular professional development sessions that include all team members will be developed. When new schools enter the program, staff from all the sectors involved could participate in a program about trauma-informed practice that has already been developed for other purposes by the Department of Education and Training (DET). Where required, medical staff could access additional training to allow for the confident assessment and management of paediatric and youth health issues. Additionally, regular professional development sessions have already been initiated to allow the expertise and challenges faced by the different sectors to be highlighted. These type of sessions are described in the literature as being a mechanism for promoting respectful and supportive relationships within multidisciplinary teams while helping the team develop a common language [68,73]. Furthermore, an incremental approach is required when scaling up. This allows processes to be reviewed and improved upon, which is a more acceptable approach to most multidisciplinary teams [68,70,74].

Professional integration can be stimulated by financial incentives [39,75]. The Kalgal Burnbona team have developed novel co-funding options with schools. For example, at NS, the school had funded a community paediatrician, social worker, speech pathologist and occupational therapist through individual contracts. This created an exemplar for the processes required to form contracts and accounts between schools and SLHD for future replication.

#### Micro Level: Clinical Integration

At the micro level of integrated care is clinical integration. Clinical integration refers to how well the process of delivering care to individual patients works [65]. For NS, and when scaling up, to provide holistic child and family centred care the model requires representation from the health, education and social care sector. For NS this was provided by a paediatrician, social worker, youth nurse and school counsellor. The team worked closely together to establish processes and to develop an understanding of workflow. For example, during the multidisciplinary clinical assessments the youth nurse often saw the student separately to establish rapport and assess for psychosocial factors. Simultaneously, the paediatrician and social worker would take a medical and social history from the family [76]. The team would then summate findings with the student and family, taking a partnership approach to prioritising needs and developing recommendations [78]. This type of practice differs to mainstream clinical services which tend to be disease-focused rather than person-focused [65]. SBIC provides innovation in this aspect, which is particularly relevant for socially disadvantaged populations who often have needs that span across multiple services. A person-focused approach to clinical integration can improve wellbeing and facilitate the continuous, comprehensive, and coordinated delivery of services at an individual level [65,77].

#### Macro Level: System Integration

At the macro level of integrated care, the guiding principles behind an initiative are used to drive the structures, processes and techniques that transect multiple sectors and contribute to health and wellbeing across the life-course. For the Kalgal Burnbona framework, the model of care which was designed in collaboration with schools and the community, steered health services away from mainstream settings to schools, where they were more accessible to those who needed them. As a result of this community ownership, SLHD have since partnered with the Student Wellbeing Unit within the DET to work towards aligning processes and to collaborate so that the model can be scaled up. Furthermore, a community of practice across New South Wales (NSW) is being led by the Kalgal Burnbona team to facilitate cross-sector partnerships.

#### Functional integration and normative integration

Finally, functional and normative integration spans the micro, meso and macro level and ensures connectivity within a system [65]. Functional integration relates to the key mechanisms involved in financing, information, and management systems e.g. human resources, strategic planning, and quality improvement [68,75]. In developing a framework for Kalgal Burnbona, MOUs across sectors as well as procedural documents to guide multidisciplinary teams at individual schools have been formed. These documents provide structure but are not prescriptive in nature because a degree of flexibility is required to meet the needs of individuals and communities [68]. The Kalgal Burnbona team have produced a logic model and manualised how to set up and evaluate SBIC to support and coordinate service delivery, accountability and decision-making between organisations and professionals [65].

Normative integration is another dimension that connects the micro, meso and macro levels of integrated care systems. This is a less tangible element of integrated care and refers to the underlying values and beliefs that are shared across the system and which allow cohesion and consistency [39,40]. The Kalgal Burbona framework is in the early stages of formation. As such, normative integration is being developed at a micro and meso level by the community and cross-sector teams involved in shaping the model. This includes regular meetings to develop the model and its evaluation as well as case discussion meetings [63]. Teams also develop close working relationships during the process of clinical assessment itself [78]. Furthermore, the ‘leaders committee’ is responsible for developing and maintaining the mission and vision of the program, which is filtered into the culture of each organisation [65].

### Kalgal Burnbona Framework

**Figure 3:**
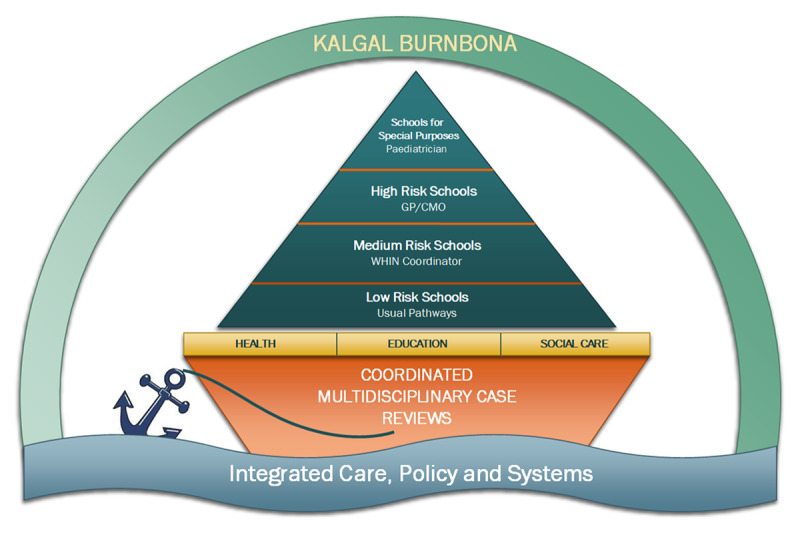
Kalgal Burnbona Model of Care.

Considering the above frameworks and knowledge derived from the mixed methods evaluation of NS, a proposed framework called Kalgal Burnbona will be described. Kalgal Burnbona is a framework that provides structure for SBIC but builds in flexibility so that the model can be adapted to the needs and resources available within a community. Focusing on SLHD, the framework firstly categorises schools by priority levels i.e. low priority, medium priority, high priority, and special education tiers. This is visually represented in Figure 3 where schools are represented by a sail of a boat. Moving from the base of the sail to the top, as the priority level of schools increase so does the level of support required. The main body of the boat represents the importance of multidisciplinary case reviews held by the health, education and social care sectors. These joint and collaborative sessions allow for information to be shared across sectors, for responsibilities to be assigned to individuals, and opportunities to discuss outstanding or new issues for families. The waves underneath the boat represent the need for the model to adapt to the natural ebbs and flows inherent of working with individuals, families and communities as well as across sectors. The model is ‘anchored’ by the concept of integrated care, policy and systems.

Categorisation of schools into priority levels was based on a combination of community knowledge and national indexes of socioeconomic advantage [79]. More specifically, schools within SLHD can be stratified according to risk by using the following information: Index of Community Socio-Emotional Advantage value (ICSEA), Index of Relative Socio-Economic Disadvantage (IRSD) ranking for surrounding suburbs to school, intentional self-harm hospital presentation within the local government area (LGA), interpersonal violence-related hospital presentation within the LGA, percentage of Aboriginal students and percentage of culturally and linguistically diverse (CALD) students. Community knowledge was drawn upon to see if there was agreement regarding how each school was prioritised. This included discussions with senior team members from community paediatrics, the HHAN integrated care team and youth health services. This additional information was valuable because many areas within central Sydney have undergone redevelopment. So, while these areas would rate as being economically ‘advantaged’, there were certain populations and schools within those areas which experience high levels of disadvantage (Table 1).

**Table 1:** Characteristics of Schools within the SLHD Community Paediatrics Team.


STUDENT CHARACTERISTICS	SCHOOL						

	SCHOOL A	SCHOOL B	SCHOOL C	SCHOOL D	SCHOOL E	SCHOOL F	SCHOOL G

**Number of students (n)**	1034	34	282	412	27	288	514

**Female (n %)**	448 (43%)	6 (18%)	147 (52%)	194 (47%)	6 (22%)	116 (40%)	261 (51%)

**Male (n %)**	586 (57%)	28 (82%)	135 (48%)	218 (53%)	21 (78%)	172 (60%)	253 (49%)

**Aboriginal students (%)**	17%	24%	18%	9%	52%	4%	0%

CALD background*(%)	57%	43%	54%	55%	25%	81%	97%

ICSEA score and centile**	1037 (64^th^)	934 (18^th^)	1018 (55^th^)	1051 (70^th^)	851 (4^th^)	985 (39^th^ )	1094 (84^th^)

SEA Distribution***							

– **Bottom quartile**	19% (25%)	46%	25%	15%	63%	42%	8%

– **Middle quartile**	19% (25%)	23%	17%	22%	24%	27%	16%

– **Middle quartile**	28% (25%)	16%	26%	31%	9%	24%	37%

– **Top quartile**	34% (25%)	16%	32%	32%	4%	7%	38%

**IRSD^**	10	9	6	5	6	3	6

**1 = lowest**							

**10 = highest**							

**Intentional self-harm within LGA (per 100,000)**	82.4	82.4	82.4	88	82.4	51.8	51.8

**NSW Average 83 per 100,000^^**	Lower than state average	Lower than state average	Lower than state average	Higher than state average	Lower than state average	Lower than state average	Lower than state average

**Interpersonal violence with LGA**	51.6	51.6	51.6	45.2	51.6	53	53

**NSW Average 58.2 per 100,000^^^**	Lower than state average	Lower than state average	Lower than state average	Lower than state average	Lower than state average	Lower than state average	Lower than state average

**Agreed Vulnerability**	Yes	Yes	Yes	Yes	Yes	Yes	No

**Overall Priority Level**	High Risk	School for Special Purposes	High Risk	High Risk	School for Special Purposes	Medium Risk	Low Risk


*CALD = culturally and linguistically diverse. ** ICSEA = Index of Community Socio-Emotional Advantage. The Australian average is 1000 [79]. *** SEA = Socioeconomic Advantage. The Australian average is 25% for each quartile [79]. ^IRSD = Index of Relative Socio-Economic Disadvantage (IRSD) ranking for surrounding suburbs to school [82]. ^^Data from 2019/2020 [83]. ^^^Data from 2019/2020 [84].

By categorising schools using priority levels, we have established a system for understanding what type of multidisciplinary team will be needed by each school. Schools for Special Purposes (SSPs) ‘provide specialist and intensive support in a dedicated setting for students with moderate to high learning and support needs’ [80]. SSPs specifically cater for students with intellectual disability, mental health issues, autism, physical disability, sensory impairment, learning difficulties or behaviour disorders [80]. At the time of describing this model, only SSPs for students experiencing externalising behavioural issues were included. The nature and complexity of students attending these SSPs will mean that a paediatrician skilled in these areas will be needed to form the health arm of the team.

High priority schools require medical staff with specialised skills in managing paediatric behavioural and developmental skills. These skills can be provided for by paediatricians, general practitioners (GPs) and Career Medical Officers (CMOs), with the provision of additional training or upskilling where required. The health arm for medium priority schools can be provided by other health staff. For example, in NSW a Wellbeing and Health In-Reach Nurse (WHIN) coordinator program was established in 2021. The WHIN program is a partnership between NSW Health and DET whereby a WHIN coordinator is positioned at selected schools across the state. Within SLHD there are four WHIN coordinators situated across 16 primary and secondary schools. The WHIN coordinators work closely with the school’s wellbeing, learning and support teams as well as local health and social services to support students and their families on a wide range of health and wellbeing issues [81]. Potentially, WHIN coordinators could work within the Kalgal Burnbona framework and be supported by community paediatricians. All low priority schools can access existing health and social care pathways within SLHD. Table 1 describes the characteristics of de-identified schools within the SLHD community paediatrics team.

In addition to a health arm, all levels within the Kalgal Burnbona priority system require an education arm and a social care arm. The education arm will generally be represented by a school’s learning support team (LST) which typically consists of the school counsellor and assistant principal. Provision of social care will vary across schools and will be based on availability of resources and collaborating with partnering services such as youth services and NGOs.

To strengthen the model, all staff will participate in regular professional development sessions. Furthermore, the social workers involved in Kalgal Burnbona will form a clinical supervision group with senior clinicians from HHAN.

### An Example of Scaling Up: Alexandria Park Community School (APCS)

**Figure 4:**
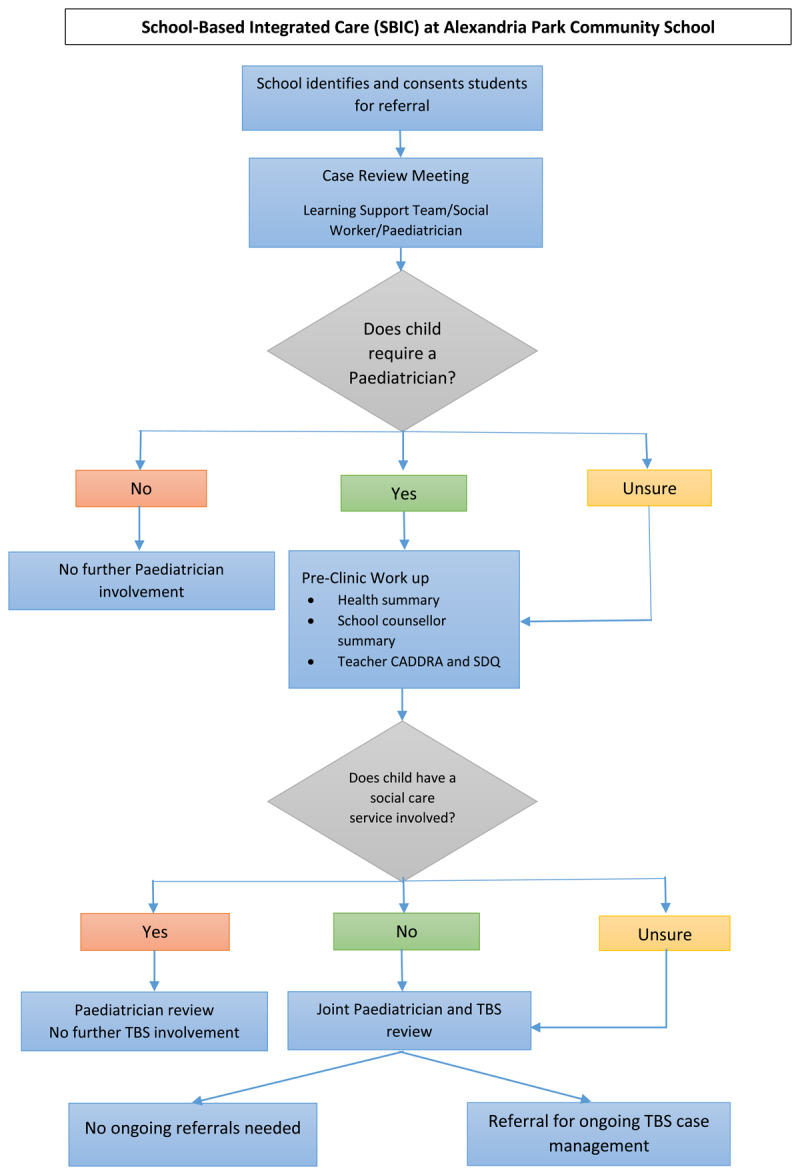
Flowchart showing the model of care adapted for Alexandria Park Community School. TBS = The Benevolent Society social worker.

APCS will be used to describe how the original NS model can be adapted to meet the needs of individual schools and communities (Figure 4). We propose that at each SBIC there is a dedicated ‘School Health Team’ consisting of representatives from the health, education and social sector. For APCS, the health sector will be represented by a community paediatrician, while the education representatives will include the school counselling team, the deputy principal and an Aboriginal Education Officer (AEO). Social care will be provided by The Benevolent Society (TBS), which is a NGO providing integrated support services for children, young people, families, people with disability, older Australians and carers [85].

The School Health Team will receive referrals from the LST. At the time of referral, a named representative from the education team will obtain consent from the student’s parent/caregiver for information to be shared by the services involved. A list of referrals will be reviewed at a ‘Case Review Meeting’. When a child is already known to a health or social service, they will be connected with or discussed with that existing service to see if an assessment at the SBIC is required.

If the child does require a multidisciplinary SBIC assessment, information will be gathered prior to the assessment. This includes a health summary, school counselling file summary and a teacher report consisting of a Canadian ADHD Resource Alliance (CADDRA) report and a SDQ [86,87]. The child will then be seen by a health professional and a TBS social worker. Where possible a member from the education team will also be present. After the assessment, the primary concerns will be discussed within the team and recommendations actioned by the appropriate team member. Families will be supported by the social worker to navigate referral pathways or to help meet other outstanding issues.

At the following Case Review Meeting, the student’s progress and any outstanding matters will be discussed. This process will be ongoing and documented. When a student no longer needs review at this meeting they will be taken off the list for discussion.

## Discussion

Integrating care for local implementation of novel strategies such as SBIC is complex and involves overcoming many barriers in the health and social care systems [65,68,88]. Kalgal Burnbona is a hypothetical framework for scaling up SBIC across NSW based on a pilot site called NS. SBIC has been shown to improve access and engagement with health services in a culturally-safe manner that is acceptable to schools, communities and professionals [64].

We propose a tiered approach to the equitable delivery of services by developing a prioritisation system for schools. Depending on the priority level, representatives from the health, education and social sectors will form a School Health Team that assesses and manages the needs of children and their families. At this micro level, schools and stakeholders need a degree of preparation before moving towards implementation of the model of care. This involves an understanding of the goals, processes and resources required as well as a practical understanding of the roles of each sector and what outcomes can be expected [71]. For multidisciplinary teams to build cohesion, creating guidelines does provide structure to govern roles and responsibilities. Such guidelines do however need to be managed flexibly to avoid creating further barriers to accessing care [68,70]. Communication between the teams and interprofessional interactions are important in developing effective teams [70,89]. Within each school, it is proposed that the School Health Team meet regularly to discuss cases and processes. Amongst the wider Kalgal Burnbona team, regular professional development sessions will be established to facilitate knowledge sharing and to provide networking opportunities [90,91].

At a meso level, an overarching ‘leaders team’ will provide oversight of the initiative while local clusters of schools will form community reference groups. These teams will consist of stakeholders from each sector and the community, promoting shared responsibility for the program. Policy and guidelines will provide further integration at a meso level e.g. MOUs, procedural documents. Consistent evaluation measures will be developed in consultation with community and stakeholders with the aim of embedding accountability across all the postulated sites.

At a macro level, the principles of community empowerment and co-design, culturally-safe, holistic, child-and family centred and accessible care are the underlying drivers of the Kalgal Burnbona framework. These beliefs have and will continue to influence decisions and processes developed to produce lasting functional and normative integration as exemplified through ongoing partnerships with DET, forming a community of practice, manualising SBIC, a logic model and an evaluation.

## Conclusion

Kalgal Burnbona serves as an example of a community-driven response to inequitable health, education and social outcomes for children and families experiencing developmental, behavioural and mental health concerns. By collaborating and building partnerships that align with the model of care consistency and transparency can be achieved. The framework described provides structure for multisector teams to work within but allows for flexibility, recognising that each community and school had its own history and needs. Resourcing the model requires creative solutions and active formation of partnerships. To bring together the various teams and schools within the model, support networks and joint professional development sessions are necessary. The Kalgal Burnbona model can be scaled up to serve a wider network of students across the state if not nationally. The initial successes of the model provide evidence for policy changes and advocacy and allows knowledge and resources to be shared.

## Key Points

- School-based integrated care (SBIC) provides a potential solution for improving access and engagement to healthcare for priority populations across New South Wales (NSW).
- Kalgal Burnbona provides a theoretical tiered approach to provide SBIC across Sydney Local Health District (SLHD) according to need.
- The Kalgal Burnbona framework aligns with Aboriginal concepts of Social and Emotional Wellbeing (SEWB) and the importance of taking a holistic approach to health.
